# IgA is the predominant isotype of anti‐β2 glycoprotein I antibodies in rheumatoid arthritis

**DOI:** 10.1002/jcla.23217

**Published:** 2020-01-22

**Authors:** Sarra Melayah, Maha Changuel, Amani Mankaï, Ibtissem Ghedira

**Affiliations:** ^1^ Laboratory of Immunology Farhat Hached University Hospital of Sousse Sousse Tunisia; ^2^ Department of Immunology Faculty of Pharmacy University of Monastir Monastir Tunisia; ^3^ High School of Sciences and Techniques of Health Tunis El Manar University Tunis Tunisia

**Keywords:** anti‐cardiolipin antibodies, anti‐β2 glycoprotein I antibodies, rheumatoid arthritis, Tunisia

## Abstract

**Background:**

The aim of this study was to determine the frequency of anti‐cardiolipin antibodies (aCL) and anti‐β2 glycoprotein I antibodies (aβ2GPI) among Tunisian patients with rheumatoid arthritis (RA).

**Methods:**

Ninety RA patients with positive anti‐cyclic citrullinated antibodies (anti‐CCP) and 90 healthy blood donors (HBD) were studied. aCL and aβ2GPI of isotype IgG, IgA and IgM were detected by ELISA.

**Result:**

The frequency of antiphopholipid antibodies (aPL) (aCL and/or aβ2GPI) was significantly higher in patients with RA than in HBD (35.5% vs 11.1%, *P* = .0001). The frequencies of aCL and aβ2GPI were significantly higher in patients than in healthy subjects (15.5% vs 5.5%, *P* = .04 and 32.2% vs 11.1%, *P* = .0005 respectively). aβ2GPI‐IgA were significantly more frequent in patients than in the control group (26.7% vs 7.8%, *P* = .0007). In patients, aβ2GPI‐IgA were significantly more frequent than aβ2GPI‐IgG (26.7% vs. 6.7%, *P* = .0003) and aβ2GPI‐IgM (26.7% vs 5.6%, *P* = .0001). In RA patients, the frequency of aβ2GPI was significantly higher than that of aCL (32.2% vs 15.5%, *P* = .008). aβ2GPI‐IgA was significantly more frequent than aCL‐IgA (26.7% vs 4.4%, *P *= .00005). The average titer of anti‐CCP in aPL positive patients was significantly higher than in aPL negative patients (170.6 ± 50 RU/mL vs 147.7 ± 51 RU/mL, *P* = .04). Significant correlation was found between aβ2GPI‐IgA and anti‐CCP (*r* = .235, *P* = .026).

**Conclusions:**

aPL and particularly aβ2GPI‐IgA are frequent in RA and are correlated with anti‐CCP.

## 1 INTRODUCTION

Rheumatoid arthritis (RA) is a chronic, inflammatory joint disease of autoimmune nature. The ethiopathogenic mechanisms involved are complex and include gut dysbiosis.^1^ RA is characterized by autoantibodies production (rheumatoid factor (RF) and anti–citrullinated protein antibody (ACPA)). RA can lead to accumulating joint damage and irreversible disability.^2^

Antiphospholipid antibodies (aPL) are a heterogeneous group of antibodies that have been associated with thrombotic or obstetrical events in patients with antiphospholipid syndrome (APS).^3^ These antibodies can occur not only in APS but also in a variety of autoimmune, malignant, and infectious diseases.^4^ In fact, the definition of clinically significant aPL positivity is not well established.^3^

The most commonly detected aPL antibodies are lupus anticoagulant, anti‐cardiolipin antibodies (aCL) and anti‐β2 glycoprotein I (aβ2GPI). In RA, several studies determined the frequency of aCL and aβ2GPI.^5^, ^6^, ^7^, ^8^, ^9^, ^10^, ^11^ However, to our knowledge, aβ2GPI‐IgA has been determined in only three studies.^7^, ^8^, ^9^ Furthermore, the frequency of aPL antibodies is not known in RA in Tunisia. So, the aim of our study is to evaluate the frequency of aCL (IgG, IgA, IgM) and aβ2GPI (IgG, IgA, IgM) in a cohort of RA patients without looking for APS.

## 2 MATERIALS AND METHODS

### 2.1 Patients

In our retrospective study, sera of 90 RA patients, with positive anti‐cyclic citrullinated antibodies (anti‐CCP), were included from the database of our Immunology laboratory. Sera were collected between 2017 and 2018 from four hospitals in the center of Tunisia. Patients were diagnosed with RA according to American College of Rheumatology/European League Against Rheumatism (ACR/EULAR).^12^

Sera of sex‐matched 90 healthy blood donors (HBD) served as normal controls. All sera of control group were tested for anti‐CCP and RF.

All sera were stored at −80°C until the use. Ethical committee of our hospital gave approval for this study.

### 2.2 Methods

#### 2.2.1 aCL assays

Serum samples were evaluated for aCL‐IgG, IgA, and IgM by using a commercial enzyme‐linked immunosorbent assay (ELISA) (Orgentec Diagnostika^®^) as we have described it previously.^13^ Results were expressed as arbitrary units with a cutoff of positivity of 10 U/mL for IgA and IgG and 7 U/mL for IgM following the manufacturer's instructions.

#### 2.2.2 aβ2GPI assays

The determination of aβ2GPI IgG, IgA, and IgM were carried out with a commercial ELISA (Orgentec Diagnostika^®^) using a purified human β2GPI as we have described it previously.^13^ Results were expressed as arbitrary units with a cutoff for positivity of 8 U/mL following the manufacturer's instructions.

#### 2.2.3 RF assays

Serum samples were evaluated for IgG, IgA, and IgM‐FR by using a commercial ELISA (Orgentec Diagnostika^®^) as we have described it previously.^14^ Results were expressed as arbitrary units following the manufacturer's instructions.

#### 2.2.4 Anti‐CCP assays

Anti‐CCP was detected by using a commercially available second‐generation ELISA (Euroimmun^®^) as we have described it previously.^15^ Results were expressed as arbitrary units with a cutoff for positivity of 5 RU/mL according to the manufacturer's instructions.

#### 2.2.5 Statistical analysis

The comparison of frequencies of aPL was performed using Chi‐square or Fisher's test. The variables were tested for normality using the Kolmogorov‐Smirnov test. To compare the mean titer of anti‐CCP between positive and negative aPL patients, we used a parametric Student's *t* test. Correlation study between aβ2GPI‐IgA and anti‐CCP was done by calculating Spearmans's correlation coefficient. A *P*‐value <.05 was considered significant.

## 3 RESULTS

The characteristics of patients and normal controls are presented in Table 1.

**Table 1:** Characteristics of RA patients and the control group

	RA patients (n = 90)	Control group (n = 90)
Sex‐ratio	1.6	1.6
(F/M)	(56/34)	(56/34)
Mean age	53 ± 15 y	37 ± 11 y
Age range	22‐83 y	20‐64 y
Positive anti‐CCP	100% (90/90)	3.3% (3/90)
Positive IgG‐RF	78.8% (71/90)	2.2% (2/90)
Positive IgA‐RF	78.8% (71/90)	0% (0/90)
Positive IgM‐RF	90% (81/90)	5.5% (5/90)

aCL and aβ2GPI frequencies are summarized in Table 2. The frequency of having any type of aPL (aCL and/or aβ2GPI) was significantly higher in patients with RA than in HBD (35.5% vs 11.1%, *P* = .0001).

**Table 2:** Frequency of aCL and aβ2GPI in patients with RA and in the control group

Autoantibodies	RA patients (n = 90)	Control group (n = 90)	*P*
aPL (aCL or aβ2GPI)	35.5% (32/90)	11.1% (10/90)	.0001
aCL (IgG, IgA or IgM)	15.5%**** (14/90)	5.5% (5/90)	.04
aCL‐IgG	8.9% (8/90)	2.2% (2/90)	NS
aCL‐IgA	4.4% * (4/90)	2.2% (2/90)	NS
aCL‐IgM	6.7% (6/90)	4.4% (4/90)	NS
aβ2GPI (IgG, IgA, or IgM)	32.2%**** (29/90)	11.1% (10/90)	.0005
aβ2GPI‐IgG	6.7%** (6/90)	3.3% (3/90)	NS
aβ2GPI‐IgA	26.7%*, **, *** (24/90)	7.8% (7/90)	.0007
aβ2GPI‐IgM	5.6%*** (5/90)	4.4% (4/90)	NS

* Comparison between aCL‐IgA and aβ2GPI‐IgA (*P* = .00005).

** Comparison between aβ2GPI‐IgG and aβ2GPI‐IgA (*P* = .0003).

*** Comparison between aβ2GPI‐IgM and aβ2GPI‐IgA (*P* = .0001).

**** Comparison between aCL and aβ2GPI (*P* = .008).

In RA patients, the frequency of aβ2GPI was significantly higher than that of aCL (32.2% vs 15.5%, *P* = .008). aβ2GPI‐IgA was significantly more frequent than aCL‐IgA (26.7% vs 4.4%, *P* = .00005).

**Figure 1:**
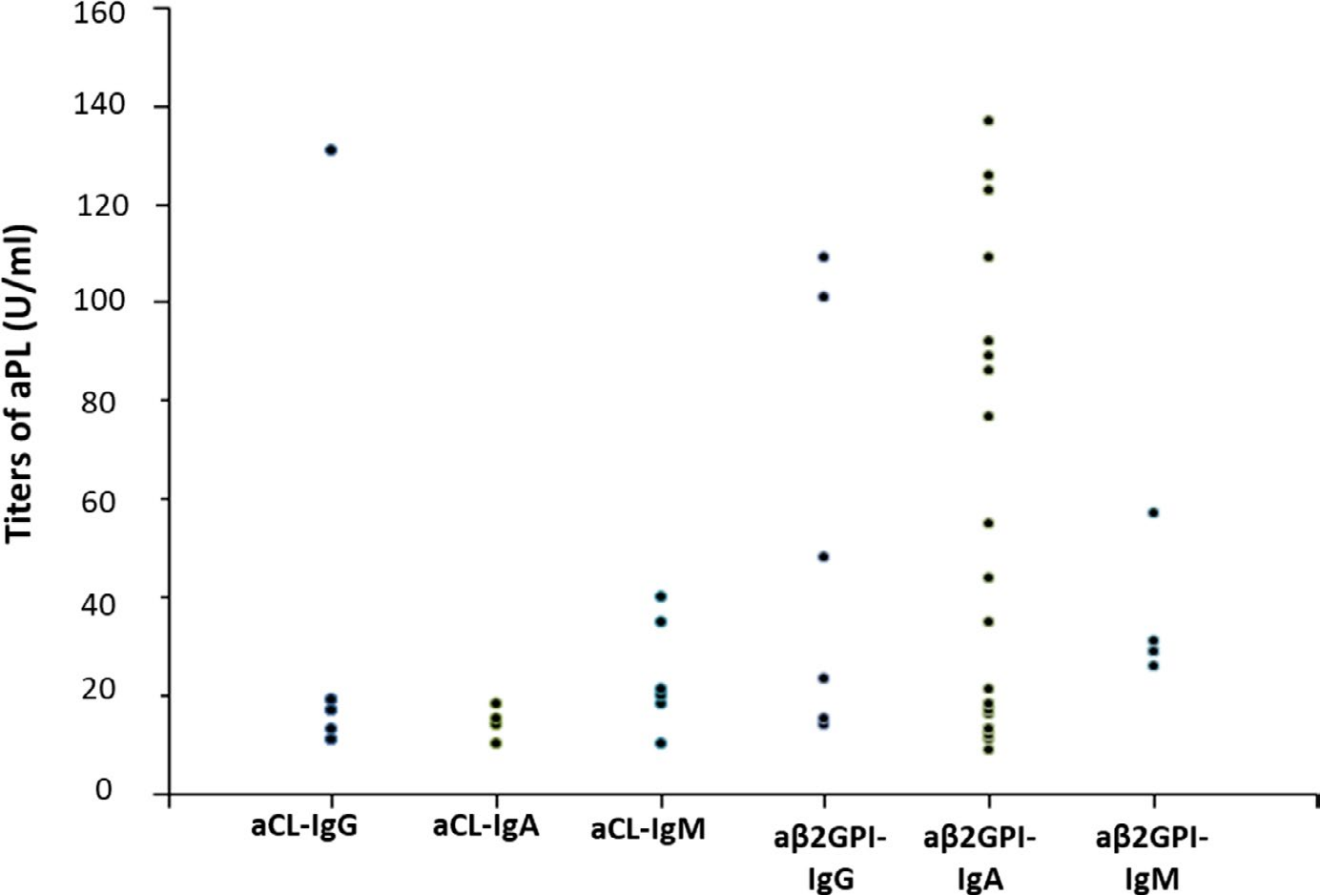
Distribution of titers of aCL and aβ2GPI in positive aPL patients

Distribution of titers of aCL and aβ2GPI in positive aPL patients is presented in Figure 1.

### 3.1 Frequencies of aCL‐IgG, IgA, and IgM

The frequency of aCL (IgG, IgA, or IgM) was significantly higher in RA patients than in controls (15.5% vs 5.5%, *P* = .04).

### 3.2 Frequencies of aβ2GPI‐IgG, IgA, and IgM

The frequency of aβ2GPI (IgG, IgA, or IgM) was significantly higher in RA patients than in the control group (32.2% vs 11.1%, *P* = .0005). aβ2GPI‐IgA was significantly more frequent in RA patients than in HBD (26.7% vs 7.8%, *P* = .0007). In RA patients, aβ2GPI‐IgA was significantly more frequent than aβ2GPI‐IgG (26.7% vs 6.7%, *P* = .0003) and aβ2GPI‐IgM (26.7% vs 5.6%, *P* = .0001).

### 3.3 Frequency of aPL according to sex

In RA patients, the frequency of aPL was not statistically different between females and males (32.1% and 41.2%, respectively) (Table 3). The frequency of aPL was significantly higher in female patients than in healthy females (32.1% vs 8.9%, *P* = .004). Male patients had a significantly higher frequency of aPL than healthy males (41.2% vs 14.7%, *P* = .02). In females, aβ2GPI and aβ2GPI‐IgA were significantly more frequent in patients than in healthy subjects (30.3% vs 8.9%, *P* = .007 and 23.2% vs 7.1%, *P* = .03, respectively). The same results were obtained for males (35.3% vs 11.8%, *P* = .04 for aβ2GPI and 32.3% vs 8.8%, *P* = .03 for aβ2GPI‐IgA).

**Table 3:** Frequency of aPL according to sex

Autoantibodies	Females			Males		
	RA patients (n = 56)	Control group (n = 56)	*P*	RA patients (n = 34)	Control group (n = 34)	*P*
aPL	32.1% (18/56)	8.9% (5/56)	.004	41.2% (14/34)	14.7% (5/34)	.02
aCL	16.1% (9/56)	5.3% (3/56)	NS	14.7% (5/34)	5.9% (2/34)	NS
aβ2GPI	30.3% (17/56)	8.9% (5/56)	.007	35.3% (12/34)	11.8% (4/34)	.04
aβ2GPI‐IgA	23.2% (13/56)	7.1% (4/56)	.03	32.3% (11/34)	8.8% (3/34)	.03

### 3.4 Association between aPL and RA antibodies

**Figure 2:**
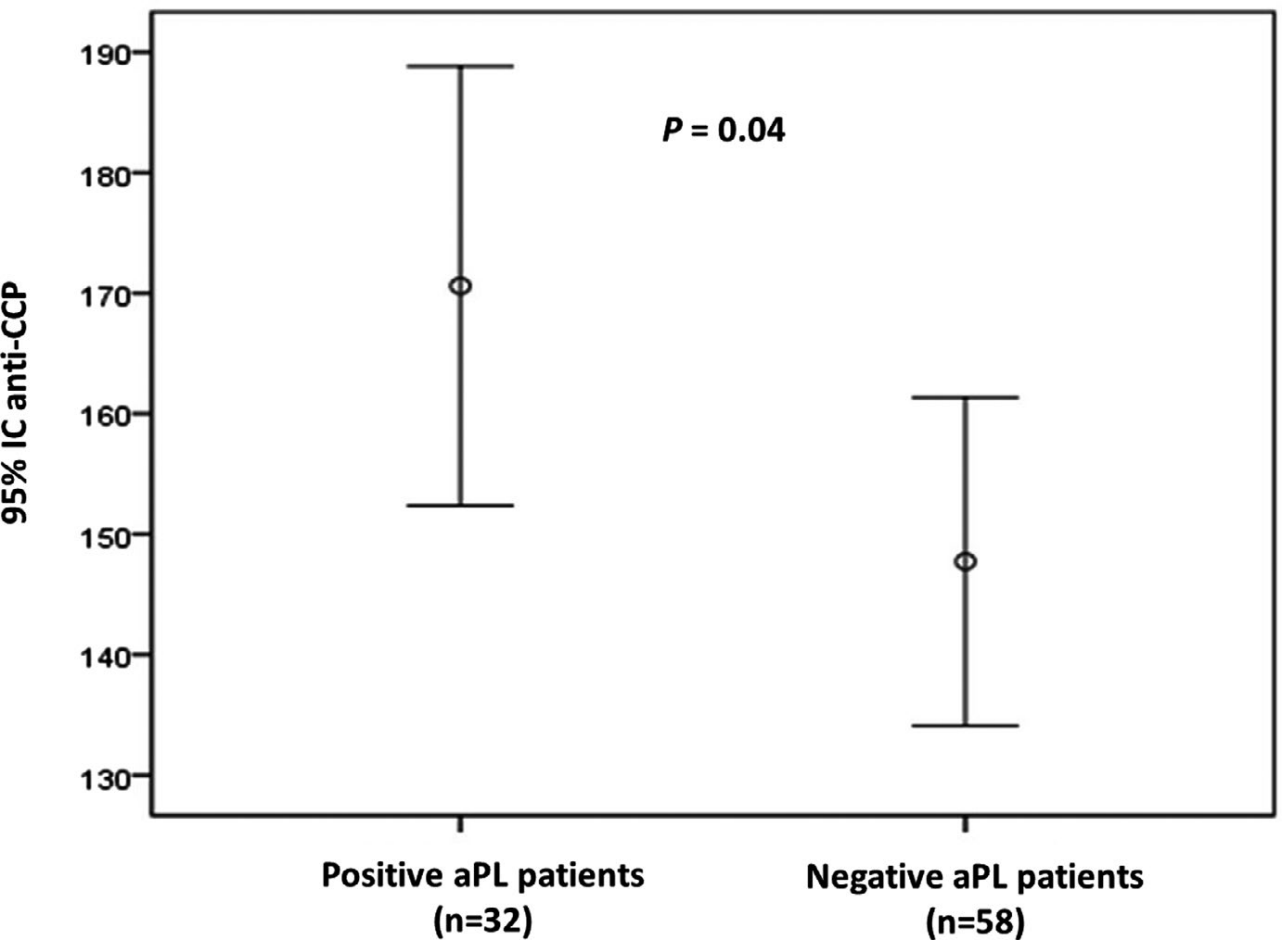
Association between anti‐CCP mean titer and aPL in RA patients

The average titer of anti‐CCP in aPL positive patients was significantly higher than in aPL negative patients (170.6 RU/mL ± 50 vs 147.7 ± 51 RU/mL, *P* = .04) (Figure 2).

No significant difference was found in the average titer of RF (IgG, IgA, or IgM) between positive and negative aPL patients.

Significant correlation was found between titers of aβ2GPI‐IgA and titers of anti‐CCP (*r* = .235, *P* = .026).

## 4 DISCUSSION

This study provides evidence for an increased frequency of aPL (aCL and/or aβ2GPI) in patients with RA compared to the control group (35.5% vs 11.1%; *P* = .0001). The frequency of aPL in our RA patients is similar to that found by Pahor et al^7^ (35.5% and 37%, respectively) and higher than those found by Ambrozic et al^8^ and Palomo et al^9^ (23% and 19.1%, respectively). The frequency of aCL in our study is similar to that of Merkel et al^10^ (15.5% and 15.7%, respectively) but lower than that of Wolf et al^11^ (32%). This discrepancy could be explained by the difference between the epidemiological characteristics of RA patients included and the methods used for aPL measurement (Table 4).

**Table 4:** Frequency of aPL in patients with RA in Literature

Authors	Number of patients	aPL (%)	aCL‐IgG (%)	aCL‐IgA (%)	aCL‐IgM (%)	aβ2GPI‐IgG (%)	aβ2GPI‐IgA (%)	aβ2GPI‐IgM (%)
Merkel et al^10^	70	15.7	11.4	0	4.3	‐	‐	‐
Wolf et a^11^	173	32	20	‐	16	‐	‐	‐
Ambrozic et al^8^	53	23	8	17	8	6	8	11
Pahor et al^7^	70	37	12.8	‐	4.3	10	25.7	2.8
Palomo et al^9^	84	19.1	8.3	0	2.4	7.2	0	4.8
Our study	90	35.5	8.9	4.4	6.7	6.7	26.7	5.6

In the present study, the frequency of aβ2GPI is similar to that found by Pahor et al^7^ (32.2% and 30%, respectively). In our RA group, IgA was the predominant isotype of aβ2GPI and its frequency is similar to that of Pahor et al^7^ (26.7% and 25.7%, respectively). The frequency of aβ2GPI‐IgA is higher than that found by Ambrozic et al and Palomo et al^8^, ^9^ (8% and 0%, respectively) (Table 4). The predominance of IgA class of aβ2GPI in our RA patients is in agreement with our previous studies on the frequency of aβ2GPI in other autoimmune diseases (Table 5).^13^, ^16^, ^17^, ^18^ Indeed, it has been reported that IgA is the predominant isotype of aPL antibodies in Afro‐Caribbeans^19^ and also in Afro‐Americans.^20^

**Table 5:** Predominance of aβ2GPI‐IgA in our previous studies

Authors	Autoimmune diseases	aβ2GPl‐IgG (%)	aβ2GPl‐IgA (%)	aβ2GPl‐IgM (%)
Mankaï et al^16^	Celiac disease	1.6	14.3	1.6
Mankaï et al^17^	Systemic lupus erythematosus	19.8	50.9	‐
Mankaï et al^13^	Primary biliary cholangitis	12.5	62.5	21.2
Mankaï et al^18^	Antiphospholipid syndrome	22	83.1	‐
Present study	Rheumatoid arthritis	6.7	26.7	5.6

Alessandri et al^21^ reported anti‐mutated citrullinated vimentin antibodies (anti‐MCV), autoantibodies of RA, in APS and we found aβ2GPI in RA. Moreover, Alessandri et al^21^ found a correlation between anti‐MCV and arthritis in APS patients and we found a correlation between aβ2GPI and anti‐CCP in RA patients. So, we tried to know if there is a similarity between these two diseases. Interestingly, dysbiosis of gut microbiota was described not only in RA^1^, ^22^ but also in APS.^23^ This dysbiosis induces not only protein citrullination^22^ but also a conformational change of β2GPI, that exposes a cryptic epitopes in domain I of β2GPI^23^ and therefore aβ2GPI synthesis.^24^

In our RA patients, we found a high frequency of aβ2GPI and a correlation between aβ2GPI‐IgA and anti‐CCP. So, the question arises: are aβ2GPI‐IgA implicated in the pathogenesis of arthritis in RA? During RA, gut microbiota dysbiosis may cause the activation of innate‐like T cells, which can be skewed toward a pro‐inflammatory state and contribute to inflamed joint tissue.^25^ Aberrant epigenetic changes (histone modifications, DNA methylation, and miRNAs) are implicated in inflammatory joints of RA.^26^ Moreover, phospholipid transfer protein is highly expressed in joints and its activity in synovial fluid is elevated and correlated with pro‐inflammatory cytokines (Il‐1 β, Il‐6) and, therefore, it may directly trigger inflammation.^27^ Surprisingly, during joint inflammation, enzymatically activated β2GPI is transformed from closed conformation to an open hockey stick‐like conformation. The resulting aβ2GPI is responsible for cartilage degradation of phospholipid bilayers and, therefore, boundary‐lubricating ability is deactivated.^28^ Moreover, through multiple mechanisms, aPL activity results not only in vasculopathy, thrombosis, and pregnancy complications but also in inflammation.^3^ So, could aβ2GPI be both the cause and the consequence of the inflammation in the synovial joints?

Gut microbiota dysbiosis^1^, ^22^ is associated with an intestinal barrier dysfunction.^29^ Alterations in gut permeability may allow intraluminal compounds entry the mucosal site, and this may be a trigger cause of an autoimmune reaction. In the gut, there is not only microbiota but also mycobiota (fungal community).^30^ Two major genera in the mycobiota were Candida and *Saccharomyces*.^31^ Because of a leaky gut, *Saccharomyces cerevisiae* arrives to the mucosa and induces the synthesis of antibodies to Saccharomyces cerevisiae named ASCA.^30^ ASCA has been described in RA.^32^

Interestingly, cross‐reactive epitopes on β2GPI and the phosphopeptidomannan part of the cell wall of *Saccharomyces cerevisiae* have been described.^33^ In the same way, we have previously demonstrated a high frequency of ASCA in patients with aβ2GPI.^18^ So could we imagine that aβ2GPI, that we have detected in RA in the present study, are ASCA and are implicated in the pathogenesis of RA? Fascinatingly, a strong similarity between the sequence of autoantigens of RA and mannan expressed by the cell wall of *Saccharomyces cerevisiae* has been described.^34^ So, ASCA could bind to citrullinated peptides or to β2GPI in joints, inducing complement activation. Another possibility is that these antibodies bind to mannan of the yeast which arrived from the mycobiota until the joint via the vascular compartment because of a leaky intestinal wall observed in RA. Surprisingly, a new model of chronic arthritis induced by mannan from *Saccharomyces cerevisiae* has been discovered. This model involves both macrophages which express mannose receptor and complement cascade.^35^

Our study presents some limitations: 1‐ It is a retrospective one, so we do not have data on clinical manifestations and correlation between aβ2GPI‐IgA and any clinical feature of RA could not be studied. 2‐ Our study lacks an experimental demonstration on a possible pathogenic mechanism of aβ2GPI in RA.

## 5 CONCLUSION

In conclusion, we found a significantly higher frequency of aβ2GPI in RA patients in comparison to the healthy subjects and we tried to explain why these antibodies are produced in RA. We could hypothesize, as said Hippocrates "all disease starts in the gut", that RA begins in the gut by: (a) Microbiota which induces joint inflammation, protein citrullination, aβ2GPI synthesis, and intestinal barrier dysfunction. (b) Mycobiota which induces synthesis of antibodies (ASCA) who recognize self antigens such as β2GPI and citrillunated proteins. In Tunisia, stress,^36^ smoking,^37^ and high prescription of antibiotics^38^ trigger gut microbiota dysbiosis and high bread consumption trigger a mycobiota rich in *Saccharomyces cerevisiae*. All these factors combined with a high frequency of consanguineous marriage^39^ could explain the high frequency of RA in our country.

## CONFLICT OF INTEREST

None of the authors have conflicts of interest to declare.
